# Investigation of *Chlamydophila *spp. in dairy cows with reproductive disorders

**DOI:** 10.1186/1751-0147-50-39

**Published:** 2008-09-26

**Authors:** Ann-Charlotte Godin, Camilla Björkman, Stina Englund, Karl-Erik Johansson, Rauni Niskanen, Stefan Alenius

**Affiliations:** 1 Division of Ruminant Medicine and Veterinary Epidemiology, Department of Clinical Sciences, Faculty of Veterinary Medicine and Animal Science, Swedish University of Agricultural Sciences (SLU), Box 7054, SE-750 07, Uppsala, Sweden; 2 Department of Animal Health and Antimicrobial strategies, Section of Antibiotics, National Veterinary Institute (SVA), SE-751 89, Uppsala, Sweden; 3 Division of Bacteriology and Food Safety, Department of Biomedical Sciences and Veterinary Public Health, Faculty of Veterinary Medicine and Animal Science, Swedish University of Agricultural Sciences (SLU), Box 7009, SE-750 07, Uppsala, Sweden; 4 Department of Bacteriology; National Veterinary Institute (SVA), SE-751 89, Uppsala, Sweden

## Abstract

**Background:**

Reports worldwide indicate high prevalence of *Chlamydophila *spp. infection in cattle. To assess the prevalence in Sweden, 525 cows in 70 dairy herds with reproductive disorders was investigated.

**Methods:**

To detect antibodies two commercially available kits were used. Moreover, 107 specimens, including vaginal swabs, organ tissues and milk were analysed by Polymerase Chain Reaction (PCR).

**Results:**

Two (0.4%) cows were seropositive in the Pourquier *Cp. abortus *ELISA. The seroprevalence with the Chekit ELISA was 28% with no difference between cases and controls. Five specimens were positive in real-time PCR and further analysed by nested PCR. *Cp. pecorum *was confirmed by partial *omp1 *DNA sequencing of the nested PCR product of vaginal swabs from control cows.

**Conclusion:**

The results suggest that *Cp. abortus *infection is absent or rare in Swedish cows whereas *Cp. pecorum *is probably more spread. They also suggest that *Chlamydophila *spp. are not related to reproduction disorders in Swedish cattle.

## Background

Chlamydia are obligate, intracellular, gram-negative bacteria that cause a wide range of diseases in humans, other mammals and birds. The two species *Chlamydophila (Cp.) abortus *(formerly *Chlamydia (C.) psittaci *serotype 1) and *Cp. pecorum *(formerly *C. pecorum*) are known to infect ruminants [1]. It has also been reported that *Cp. psittaci *may infect cattle [2-4]. In many sheep-producing countries *Cp. abortus *is known to cause Ovine Enzootic Abortion (OEA) [5]. The zoonotic potential of *Cp. abortu*s is well known and poses a threat to mainly pregnant women, handling sheep and goats [6]. Chlamydial infection in cattle has been associated with reproductive disorders including abortion, endometritis, repeat breeding, vaginitis, seminal vesiculitis, weak calves and perinatal mortality [7-11]. Moreover, symptoms such as pneumonia, conjunctivitis, enteritis, polyarthritis and encephalitis have been reported [12-14]. It has been suggested that both *Cp. abortus and Cp. pecorum *are ubiquitous in cattle [10,15,16].

Reproductive disorders and infertility are major causes of culling in dairy herds. The diagnostic rate of abortions is usually below 35% [17,18]. In Sweden, 97% of all dairy herds are free of Bovine viral diarrhoea virus (BVDV) [19] and the prevalence of *Neospora (N.) caninum *infection is 2% [20]. Furthermore, Sweden is free from *Brucella abortus*, *Leptospira *spp. and Bovine herpes virus 1 (IBR/IPV) [21]. The prevalence of chlamydial infections and their effect on reproduction in Swedish cattle is unclear and has not previously been investigated. The aim of this study was therefore to investigate the prevalence of antibodies against *Chlamydophila *spp., preferably *Cp. abortus *and the occurrence of chlamydial agents in Swedish dairy herds with a history of reproductive disorders.

## Methods

### Animals and samples

Seventy dairy herds from different parts of Sweden that experienced reproductive disorders, mainly abortions, during January 2000 to December 2006 were included in this study. Herd sizes ranged from 19 to 215 cows and all herds were free of BVDV and *N. caninum*. As part of the diagnosis investigations, blood samples were collected by local veterinarians and sent by mail to the laboratory. Samples were collected from 4 to 15 cows (average 7.5, median 6), >2 years of age from each herd, except in two herds where all cows, 32 and 34, respectively, were bled. In almost all herds (61/70) samples from both cows with clinical signs (cases) and cows with normal pregnancies and parturitions (controls) were taken, and in the other nine herds only cows with clinical signs were sampled. A total of 525 animals were blood sampled: 286 cases and 239 controls. Of the 286 cases, 179 had aborted (two-thirds during the last trimester). They were bled on the same day or up to 10 months after abortion (mostly within the first 3 months after abortion). The other cases had premature parturition or parturition at full term resulting in death, stillbirth or weak neonate, repeat breeding or vaginitis. The blood samples were centrifuged at 1000 × *g *for 10 minutes and sera collected and stored at -20°C until analysis.

Vaginal swabs (Cytobrush Plus, Medscand Medical AB), milk samples, placentas and organs from aborted foetuses were also collected from some of the herds. In total 107 specimens were submitted: 43 vaginal swabs (from 31 cases and 12 controls in 12 herds), 54 milk samples (37 cases and 17 controls, in 10 herds), organs from 5 aborted foetuses in 3 herds and 5 placentas from abortions in 5 herds. Samples were stored at -70°C prior to preparation and analysis.

### Detection of antibodies to *Chlamydophila abortus*

Two commercially available *Cp. abortus *antibody detection kits were used.

The Pourquier ELISA *Chlamydophila abortus *serum verification test (Institut Pourquier) uses a recombinant fragment of an 80–90 kDa polymorphic outer membrane protein as antigen. The kit was used according to the manufacturer's instructions, where S/P% values equal or more than 100 are considered as positive for cattle.

The CHEKIT-Chlamydia enzyme immunoassay (Dr. Bommeli AG-Idexx) is based on an inactivated antigen originally isolated from a case of abortion in sheep. The test was performed according to the instructions of the manufacturer, considering corrected optical density (OD%) values > 40 as positive.

### Detection of *Chlamydiaceae *by Polymerase Chain Reaction (PCR)

DNA was extracted from vaginal swabs for PCR analysis according to the protocol by Sachse and Hotzel [22]. For tissue specimens and milk the High Pure Template Preparation kit (Roche Diagnostics) was applied, following the manual provided by the manufacturer, with a slight modification for milk samples [16].

Analyses were made by a real-time PCR, developed by Everett et al. [23] which targets the 23S ribosomal DNA and detects the family *Chlamydiaceae*. The sensitivity of the test was estimated by spiking samples prior to DNA extraction with 10-fold dilutions of *Cp. abortus *(inactivated strain S26/3 in original concentration of 3 × 10^8 ^IFU/ml, kindly provided by D. Longbottom, Moredun Research Institute, UK). The sensitivity was estimated to 1 IFU/PCR for all specimens except milk where the sensitivity was 10 IFU/PCR.

Positive samples by the real-time PCR were further analysed for species identification by a nested PCR as described by Kaltenboeck et al. [24] and modified by Sachse and Hotzel [22]. The nested PCR targets the *omp1 *gene and identifies the four species *C. trachomatis, C. psittaci*, *C. pecorum *and *C. pneumoniae*, according to the old classification.

### DNA sequence analysis of the PCR products

Amplicons from the nested PCR were purified prior to sequencing by the GFX PCR DNA and Gel Band Purification Kit (Amersham Bioscience Europe). The PCR products were then sequenced with primers 204 pecor and chomp 336 [22] and with the BigDye Terminator v3.1 Cycle Sequencing Kit (Applied Biosystems) in combination with ethanol/EDTA/sodium acetate precipitation according to the protocol of the manufacturer. Thermocycling was performed in a GeneAmp 2700 Thermocycler (Applied Biosystems). The sequencing products were subjected to electrophoretic separation and on-line detection on an ABI PRISM 3100 Genetic Analyzer (Applied Biosystems), followed by computerized sequence evaluation (BLAST search).

### Statistics

The chi-square test was used for Chekit ELISA results to calculate significance (Minitab^® ^Release 14.2, Minitab Inc.).

## Results

### Antibody assays

**Figure 1:**
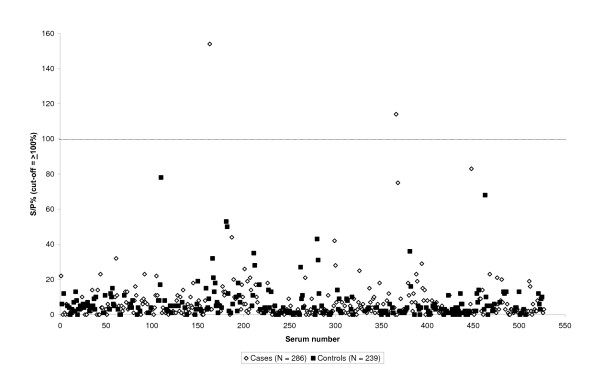
**Results of Pourquier^® ^*Cp. abortus *ELISA serology in 525 Swedish dairy cows from 70 herds with reproductive disorders, sampled during January 2000 to December 2005.** The dotted line shows the cut-off value. The seroprevalence among cases was 0.4% (2/286) and among controls 0%.

Of 525 blood sampled cows, only two (0.4%) were seropositive in the Pourquier *Cp. abortus *ELISA (S/P% values 154 and 114, respectively). They originated from two different herds. One cow had aborted in the last trimester and the other had experienced premature parturition. Most of the samples were well clustered far below the cut-off value 100%; only 6 samples (4 cases and 2 controls) had S/P% values above 60, the recommended cut-off value for sheep sera (Figure 1).

**Figure 2:**
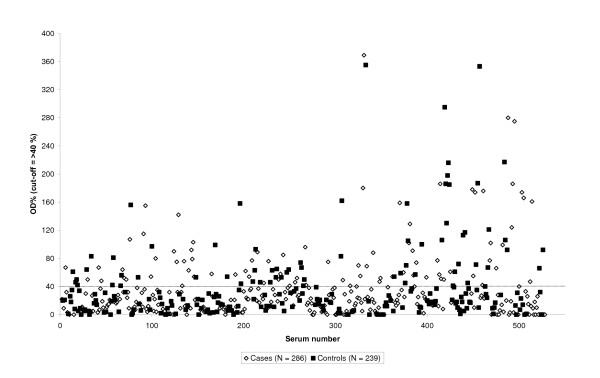
**Results of CHEKIT^®^-Chlamydia enzyme immunoassay serology in 525 Swedish dairy cows from 70 herds with reproductive disorders, sampled during January 2000 to December 2005.** The dotted line shows the cut-off value. The seroprevalence among cases was 28% (81/286) and among controls 28% (67/239).

The seroprevalence with the Chekit ELISA was 28% (148/525) and the prevalence did not differ between cases (81/286) and controls (67/239). OD% values for positives ranged between 41–369% (Figure 2). In 81% (57/70) of the herds at least one sampled cow was positive. Of the 179 cases of abortion, antibodies were detected in 55 cows (31%). The two cows seropositive in the Pourquier *Cp. abortus *ELISA were both seronegative in the Chekit assay (OD% value 3 and 21, respectively).

### PCR

*Chlamydiaceae *was detected by real-time PCR in vaginal swabs from 2 out of 12 control cows but not from any of the 31 cases. *Cp. pecorum *was detected in both swabs by nested PCR and further confirmed by partial *omp1 *DNA sequencing of the PCR product. The two cows came from different herds and were both negative in the Pourquier *Cp. abortus *ELISA and positive in the Chekit ELISA (OD% values 100 and 295).

Three more *Chlamydiaceae *positive samples were identified: a placenta from an aborting cow that was not serologically tested, and two milk samples from cows that had aborted and were negative in both antibody tests. These samples were all negative in the nested PCR, probably due to lower sensitivity of this test, and could not be sequenced. The placenta was collected in one of the herds that had a *Cp. pecorum *positive vaginal swab and the milk samples came from two other herds.

## Discussion

The results of this first investigation of *Chlamydophila *spp. infection in Swedish dairy cows suggest that *Cp. abortus *infection is absent or rare in Sweden. Only two samples were positive in the Pourquier ELISA. These might well be false positive results because both samples were negative in the Chekit assay, which is based on an antigen containing lipopolysaccharide shared by several *Chlamydiaceae*, including *Cp. abortus*. Even if we had applied the lower cut-off value used for sheep sera, only six samples would have been positive in the Pourquier ELISA, of which all except one, were negative in the Chekit ELISA. The specificity of the Pourquier ELISA has been reported to be 100% when analysed Scottish sheep documented free of OEA [25] and 90% when sera from New Zealand, a country free from *Cp. abortus*, were analysed [26].

The seroprevalence obtained in our study with Chekit ELISA probably represents antibodies against *Cp. pecorum *because it is known to infect cattle and we identified *Cp. pecorum *in vaginal swabs from two Chekit ELISA positive cows. In a recent Austrian investigation, the majority of blood sampled cows from which vaginal and cervical swabs also were taken and found *Cp. pecorum *positive by PCR, were seropositive by Chekit ELISA [27]. Further, the Chekit ELISA reacted with positive results in 26% out of 15 sera from SPF lambs immunized with *Cp. pecorum *[25]. We found no difference in Chekit ELISA seroprevalences between cows with reproductive problems, including abortion, and healthy control cows. This is in concordance with the Austrian study where no correlations between reproductive problems and Chekit ELISA or PCR positivities were found [27]. However, significantly higher seroprevalences among aborting versus healthy cows have been reported in some other studies applying the Chekit ELISA test [28,29]. Additionally, in an investigation of German herds with diverse fertility disorders, no difference in seropositivity was observed between the group of apparently healthy cows and the group of cows with abnormal reproductive performance. However, a significantly higher proportion of the cows that had aborted were seropositive [11].

The absence, or very low prevalence, of *Cp. abortus *in Swedish dairy cows could be due to the small mean herd size of 44 animals (year 2005) and that most are kept tethered during a nine-month stable period, with little contact within the herd. Further, the tradition of self-contained herds, the sparsely populated Swedish countryside with few external contacts and little movement of animals across country, give few animals a chance to get infected. The absence or very low prevalence could also be due to a generally lower infection pressure owing to lack of other infections, e.g. BVDV. Another explanation could be that *Cp. abortus *infection seems to be very low in Swedish sheep. In a recent investigation, including 800 sheep sera from different parts of Sweden, only 3 sera were positive with the Pourquier *Cp. abortus *ELISA (unpublished data).

## Conclusion

This investigation suggests that infections with *Cp. abortus *are absent or rare in Swedish cows whereas *Cp. pecorum *are probably more spread. It also suggests that *Chlamydophila *spp. is not related to reproduction disorders in Swedish cattle.

## Competing Interests

The authors declare that they have no competing interests.

## Authors' contributions

ACG drafted and rewrote the manuscript, carried out the PCR and serology analysis, interpreted the results and performed the statistical analysis. CB and SA conceived and designed the study, and participated in its coordination. SE implemented the PCR systems and carried out the sequencing analysis. KEJ participated in the sequencing analysis. All authors participated in the design of the study and have been involved in revising the manuscript.
